# Public Perspectives Around Prenatal Screening of Chromosomal Abnormalities: A Focus Group Study Comparing Metropolitan and Rural/Regional Areas in Australia

**DOI:** 10.1111/ajo.13935

**Published:** 2025-02-07

**Authors:** Amber Salisbury, Hovea Winston, Alexis Johnson, Alison Pearce, Kirsten Howard, Sarah Norris

**Affiliations:** ^1^ Menzies Centre for Health Policy and Economics University of Sydney Camperdown New South Wales Australia; ^2^ The Daffodil Centre The University of Sydney, a Joint Venture With Cancer Council NSW Sydney New South Wales Australia; ^3^ University of Sydney Medical School Dubbo New South Wales Australia; ^4^ Sydney School of Public Health The University of Sydney Sydney New South Wales Australia

**Keywords:** cell‐free DNA screening, focus groups, genetic testing, genomics, non‐invasive prenatal testing, prenatal diagnosis, prenatal screening, qualitative research

## Abstract

**Background:**

The widespread and rapid adoption of private payments for non‐invasive prenatal testing (NIPT) in Australia has introduced complexities to the decision‐making process for the public regarding prenatal screening. NIPT has the potential to be a useful screening tool, but concerns have been raised about its cost, the psychological consequences of testing and the information available to support informed decision‐making.

**Objective:**

To explore the attitudes, values and beliefs around prenatal screening in Australia, and how perspectives may differ between people living in metropolitan locations versus rural/regional locations.

**Materials and Methods:**

Three focus groups were conducted in New South Wales (NSW), Australia. Participants (*N* = 25) were recruited by a market research group. Focus groups took place face‐to‐face in metropolitan and rural/regional areas, and online via videoconference. Discussions were transcribed and analysed thematically.

**Results:**

Participants generally expressed interest in undertaking prenatal screening but held misconceptions about the purpose of NIPT (i.e. screening, not diagnosis) and the conditions assessed. There were varied opinions among participants on expanding the scope of screening: some felt additional information provided reassurance, whilst others thought it would increase stress due to the decreased accuracy. People living in rural/regional areas had greater concerns over access to screening (cost, wait times and distance) than people living in metropolitan areas.

**Conclusion:**

Our findings demonstrate different approaches are needed to improve understanding of NIPT (to ensure informed consent), and to improve access to NIPT for people living in rural/regional areas. The pre‐test information needs to account for the range of perspectives observed across geographic locations.

## 1 Introduction

Prenatal screening is a routine part of pregnancy care in most countries [1, 2]. Traditionally, combined first‐trimester screening (cFTS) has been used to offer probability‐based information on the three trisomies (T21, Down syndrome; T18, Edwards syndrome; T13, Patau syndrome) in the fetus. This approach involves ultrasound measurements of fetal nuchal translucency (NT), and the evaluation of maternal serum biochemical markers, including ß‐hCG and PAPP‐A. More recently, non‐invasive prenatal testing (NIPT) has been introduced. NIPT uses cell‐free DNA (from the fetus) to circulate in maternal plasma. NIPT has a higher detection rate and lower false positive rate for the common trisomies compared to cFTS [3]. In addition, expanded NIPT can detect a broader range of conditions, including microdeletions and duplications, and sex chromosome conditions, although with lower accuracy than when used for the three trisomies [4, 5].

There has been a widespread and rapid adoption of NIPT internationally, using different funding mechanisms [2]. Several countries have incorporated NIPT into their publicly funded national screening programs, including Belgium, Netherlands, Denmark and the UK [2, 6]. In the United States, it is covered by state or private health insurance schemes. In Australia, cFTS is publicly subsidised but NIPT is not. An Australian government consideration in 2019 accepted that a model of the universal offer of NIPT (for trisomies 21, 18 and 13) as a first‐line test was clinically efficacious and superior to cFTS, but it was not cost‐effective compared to cFTS [7]. It was acknowledged that determining the clinical and economic impact of including NIPT on the Medicare Benefits Schedule (MBS; a listing of the health services subsidised by the Australian government) was difficult and was likely to be influenced by the local availability of biochemical testing and first‐trimester ultrasounds (for cFTS), especially in rural and regional areas of the country. This led the government advisory committee to consider the possibility of an MBS item for NIPT which is “limited geographically to women in rural and remote areas”.

Numerous studies have explored the complexities and ethical issues raised by the introduction of NIPT. A recent systematic review found an overall desire among pregnant people for increased access to NIPT, although concerns were raised about the financial cost of the test, anxiety surrounding the testing process and the importance of informed decision‐making [8]. In Australia, surveys involving women who have undergone NIPT have generally reported positive experiences, but the majority of these women were of high socioeconomic status (SES) and lived in metropolitan areas [9, 10]. Whilst clinicians view NIPT as a useful and high‐quality screening test for the common trisomies, they have concerns about how NIPT has been introduced here [11, 12]. Further qualitative research is needed to understand the views of the Australian public, which are known to differ to clinician views [13].

This study uses qualitative methods to gain an in‐depth understanding of the attitudes, values and beliefs of Australian adults around prenatal screening. This is the first study to explore whether perspectives around prenatal screening differ between people living in metropolitan locations versus rural or regional locations.

## 2 Materials and Methods

### 2.1 Study Design

This qualitative study involved focus groups with adults of childbearing age interested in pregnancy. Focus groups were chosen as they enable the exploration of a variety of different perspectives and experiences through dynamic discussion that allows participants to share and compare their views with others [14]. The study was approved by the University of Sydney Human Research Ethics Committee [2023/170].

### 2.2 Setting and Participants

Three focus groups with a total of 25 participants were conducted in various geographic locations across NSW. Focus groups were conducted in the following locations (FG1) central Sydney (*n* = 9); (FG2) Dubbo (approximately 400 km Northwest of Sydney) (*n* = 6); (FG3) Online via videoconference (*n* = 10), recruiting participants from rural/regional areas in NSW. FG1 will be referred to as metropolitan, and FG2 and FG3 will be referred to as rural.

#### 2.2.1 Inclusion and Exclusion Criteria

The inclusion criteria were as follows:

- Adults (aged 18–45 years old) based in Australia, who have been pregnant in the last 4 years or are interested in becoming pregnant, or
- Partners of adults who would meet the definition of group A, but whose actual partner may or may not be a participant in the study.

Participants were not able to participate if they could not speak English.

The criteria were developed in consultation with an expert advisory group, which included three obstetricians, a genetic counsellor, a midwife, an ethics researcher working in prenatal screening and a person with a lived experience of NIPT.

### 2.3 Participant Recruitment

A market research group, Taverner Research, recruited participants using quotas to ensure variation across age, gender and education. Information about rurality, English as a second language and experience with prenatal screening were also captured.

Taverner Research sent potential participants an invitation pack, containing an invitation letter, participant information sheet and consent form. All participants provided consent prior to participation and were compensated for their time with an AU$75 gift card.

### 2.4 Conducting the Focus Groups and Discussion Content

A rapid literature review was performed to understand the broad scope of features which might impact on views around prenatal screening. Details and results can be found within the Supporting Information. Consultation with the expert advisory group was used to identify priority features for discussion within focus groups (see Table 1).

**TABLE 1:** Features of prenatal screening that were discussed within focus groups.

Priority features
**Features of the test and test results**
What the test covers (common trisomies, sex chromosome conditions, other rare conditions, sex)
Number of individuals correctly diagnosed
False positive results
False negative results
Inconclusive results
Maternal incidental findings
**Features of the process of being screened**
Cost
Wait time
Travel distance to appointments
Who provides the information before the test
How the results are delivered
Who delivers the result

Focus groups were facilitated by three members of the research team (AS, AJ, HW). One researcher (AS) moderated each focus group according to the discussion guide (see Supporting Information) and a second researcher (AJ or HW) took notes. The discussion guide contained the following: background, warm‐up questions and discussion based on the priority features (noting participants were given the opportunity to raise additional features). The scope of conditions was presented to participants as follows:

- The three common trisomies (Down syndrome, Edwards syndrome, Patau syndrome)
- Sex chromosome conditions (using Turner's syndrome as an example)
- Other rare conditions

Participants were presented with two possible options:

- A test that includes the three common trisomies
- A test that includes the three common trisomies, sex chromosome conditions and other rare conditions (referred to as expanded NIPT)

The PowerPoint slides presented to participants prior to beginning the focus group discussions can be found in the Supporting Information. This content was created in collaboration with and approved by the expert advisory group.

### 2.5 Data Capture, Coding and Analysis of Qualitative Data

Focus groups were audio recorded, transcribed verbatim by a member of the research team (AS) and analysed thematically using QSR NVivo10 qualitative data management software. Two researchers (AS, HW) independently coded the transcripts taking a mixed deductive/inductive approach [15, 16, 17]. The transcript data were coded according to broad top‐level categories that matched the discussion guide. We then searched for patterns and ideas within the top‐level categories and further coded the data into themes and sub‐themes, which were developed inductively. Agreement between coders was high and discrepancies were discussed until a consensus was reached (AS, HW). Themes were categorised into the following: attitudes (evaluative judgements individuals hold about various features of testing), beliefs (representations of knowledge that individuals accept as true or hold to be the case) and values (represent what individuals consider important or desirable in life) [18].

## 3 Results

### 3.1 Participants

Twenty‐five participants were recruited, with their characteristics shown in Table 2.

**TABLE 2:** Characteristics of participants.

Characteristics	*N*. participants (%)
**Location**	
Metropolitan	9 (36)
Rural/regional	16 (64)
**Gender**	
Female	17 (68)
Male	8 (32)
**Age**	
18–25	3 (12)
26–30	9 (36)
31–35	3 (12)
36–40	8 (32)
41–45	2 (8)
**Language**	
Speaks only English at home	16 (64)
Speaks English and another language at home	9 (36)
**Education**	
High school	3 (12)
Trade/certificate/diploma	10 (40)
Undergraduate degree	7 (28)
Postgraduate degree	5 (20)
**Interest in pregnancy**	
Pregnant within the past 4 years (or partner)	15 (60)
Interested in becoming pregnant (or partner)	17 (68)

### 3.2 Themes

Six themes emerged from the focus groups. Three themes were categorised under attitudes: (1) Access to Health Care Providers and Services; (2) Delivery of information and results; and (3) Pros and cons of expanded NIPT. Two themes were categorised under beliefs: (4) Misunderstanding and lack of awareness and (5) Views of disability. One theme was categorised under values: (6) Value of NIPT. Selected themes and sub‐themes are described in more detail below, with additional sub‐themes and quotations in Table 3.

**TABLE 3:** Additional themes and/or quotations identified within focus group transcripts, by geographical location.

Themes	Illustrative quotes
**Attitudes**	
*1. Access to health care providers and services*	
Variable impact of cost as a barrier	*Metropolitan*
	FG1, female “[In response to another participant stating they would pay for NIPT] Yeah, I think yes, we can cut down on other things, because the well‐being [of the child] is very important.”
	FG1, female “I would pay to test for it all [conditions in expanded NIPT]”
	*Rural*
	FG 2, female “And I think when it comes to cost, it really depends on like people's personal circumstances cause I would never have been able to afford a 400 and whatever test like it would, that's that would have been my entire Centrelink for like 4 weeks back then. Obviously now I have a job. A full‐time job and I'm in a very different place than I was 12 years ago that… Even like if I thought OK, maybe if we even just look back five years ago, I still wouldn't have been able to afford that, like it's only just now that I'm like, OK, yeah. I'm getting a little bit older, I'm in my 30s now so there's other factors that are now at play and would make it the cost worth it, yeah.”
	FG 2, female “Another part of the cost thing is having to go to the doctors, paying for that doctor's trip, getting the test done, paying for that test, going back to the doctor's, paying for the doctor to tell you what the results are. I know that part really hurts.”
	FG 2, female “Because I feel if I didn't have money, I would just want to do it old school.”
	FG 2, female “What do we do? You know, you think. What do we do? Do we come up with this money to get this?”
Wait time was a bigger concern for those likely to experience longer delays	*Metropolitan*
	FG1, male **“**OK, so obviously you want to get it as early as possible.”
	FG1, female “I'm still the same decision as in don't really mind because it's such a like a short 11 to 18 weeks anyways, so I'm still the same decision, don't really care.”
	*Rural*
	FG 2, female “Yeah, I'm just thinking like sometimes it takes a month to get into. So if it was restricted you would have to go to Sydney”
	FG 2, female “Having weeks between when the doctor is available and when you done the test makes me a bit worried.”
	FG2, female “What if there was that significant gap and sometimes here you can wait three weeks for a GP appointment to open up. So it's like you wouldn't be able to see your doctor. And you've had this test 3 weeks ago, and now all you're doing is having anxiety and panic attacks because you don't have that information. You know, just like, constantly thinking about it”
Travel to testing: rural vs. metropolitan challenges	*Rural*
	FG2 female, “I'm from Wilcannia. That's [travel to testing] a huge problem. So yeah, that would have been a factor.”
	FG2, female 1 “If you are getting flown out with RFDS, they're gonna send you to Adelaide for testing.” [Would you go to Adeliade to have the test] FG2, female 1 “In my current position, yes, I can understand how a lot of people would not.” FG2, female 2 “Probably not me”
	FG3, male “[what would worry you] the costs and travel distance appointments, because I live in a regional area, so do my wife and I need to travel? How often in a series of periods of time? So yeah, I think those are my major considerations”
*2. Delivery of information and results*	
Personal preference determines health professional type	*Metropolitan*
	FG1, male “I think I would prefer a GP, just because I think they'd be they'd be more personally and might if it's bad news or news you weren't expecting, they might be a bit more, you know, sensitive to your situation.”
	FG1, female “I prefer for the obstetrician. Because he or she is the expert in the field and would be in a better position to provide the information than a GP.”
	Metropolitan female “…there are heaps of people out there, so just get it from everyone.”
	Metropolitan female “I think if the person you're seeing is good and specialised in all that, I'd probably be willing to travel, you know longer, within reason.”
	*Rural*
	FG3 female “[In response to the above comment] Someone else could just give you the news, like a health nurse or something. Sometimes here you can wait three weeks for a GP appointment to open up.”
	FG2, female “I think so as well for myself, it doesn't matter who delivers the result, even if it comes from a GP or an OBGYN. I'll be doing my own research anyways.”
	FG2, female “As long as they probably know the information and they got the right information, then I would accept the info as long as they're they've got the info right with me.”
	FG2, female “Like they've just gotta, they've just gotta tell you exactly what's on their little piece of paper. Not being a doctor, I have no idea. But like, it's just having someone, even if it's like a health nurse or someone else like, knows and understands the information.”
	FG3, female “I think if you've had prior pregnancies with maybe some concerns, then maybe the generic counsellor might be the way to go. But if everything's kind of sailing along smoothly, then I think that would be a scary extra step if someone suggested to see a genetic counsellor to me anyway.”
	FG3 female “I feel like GP would have been good, because where I am, you don't go to the obstetrician until you're 12 weeks or 13 weeks. So it's like he kind of expected me to already know, because that's when you get the testing done.”
Lack of trust in health care professionals to be sensitive	*Metropolitan*
	FG1, male “So like, you know, coming from a bloke's point of view. That, you know, doctors and obstetricians like, they study for a long time, but maybe not in the delicacy of sensitivity. And that poor girl was, like, you know, communicated to poorly. So I think for me, if there is an issue, it should not be negative, not the result, how it's communicated. Yeah. So just some more training around like sensitivity.”
	*Rural*
	FG2, female “I had to change my doctor because I like I felt a bit more comfortable with another doctor, so I had to look out for like a new doctor.”
	FG2, female “There was one and I was like never coming back to you again. You're in the bin. I don't think he actually did anything wrong but I was like ‘nah’. And I don't care, I'll go and see anybody and does not matter who you are, I just want to see someone because I hate doctors and I'm only here for a very specific reason so, but yeah, this one doctor has like bad vibes.”
	FG3, female “So and sometimes and I've experienced this myself. Sometimes it's a bit feels a bit hit or miss with going into GPs, and they just trying to get people in and out, make up time”
Divergent opinions on how to receive information	*Metropolitan*
	FG1, female “Same same agree, yeah, especially like if I have some question to ask, I can get it sorted over the phone straight away, without like making another appointment, waiting time…”
	FG1, female “I think good news you take anyway. But bad news you take in person.”
	FG1, male “[In response to the question of how they would like to receive results] I'd prefer to go and see in person”
	*Rural*
	FG3, male “I think I prefer to have it via email, rather than in person. So that I can have time to collect my thoughts and compose myself, and then to really articulate my questions.”
	FG3, male “I'll prefer yeah, if it is, there is a very long wait time, yeah, I want to get the results over the phone. But I need the follow up within very short time, because face to face I need for the details, discussions.”
	FG2, female “Maybe they could look at implementing if you've got a low risk you'll hear from us when we're ready, if you're high risk we're going to call you.”
	FG2, female “Yeah, I think if it was high risk, I would like to be in person because then they'll obviously be questions, discussion about next steps and that kind of stuff, but low risk, I'll take it over the phone, yeah.”
	FG2 female, “As quick as the information can come to me, I don't care how blunt it is, or how it's given.”
	[How would you prefer to receive results?] FG3 female, In person, but it was like coupled with another appointment. FG3 male Yeah in person. FG3 male, Yeah. Likewise.
*3. Pros and cons of expanded NIPT*	
Increasing information paradox: reassurance vs. uncertainty	*Metropolitan*
	FG1, male “I think even I agree that testing for everything [expanded NIPT] should be done so we're at least prepared for what's to come.”
	FG1, female “So I think the false positive thing is kind of like a big concern, and people would probably need like more actual numbers on how accurate the results are. And then also just the stress the patient would be going through, to hear about false positive result”
	FG1, male “If you test for everything [expanded NIPT], then you can make an informed decision. How to proceed further and all that kind of stuff”
	*Rural*
	FG3, male “So I'd rather have small set of endpoints determined, but more accurate as compared to various conditions, but with less accuracy”
	FG2, female “[In response to discussion about expanded NIPT] yeah, it might encourage people to choose a test that has more scope, because then you've got a greater chance of picking up things that you might not otherwise have seen in both mother and child.”
	FG2, female “It [false positives] would be a concern for myself, because we do have family members who have quite a significant disability and I can see how much pressure it puts on my fairly old parents at the moment, and we don't want to go through that”
	FG2, female “Any information is good information”
	FG3, female “It would impact my decision [on testing for a broader range of conditions]. You want something that is quite accurate really.”
**Beliefs**	
*4. Misunderstanding and lack of awareness*	
NIPT misrepresented as a diagnostic tool	*Metropolitan*
	FG1, male “Is that the harmony test?”
	*Rural*
	FG2, female “whoever is advising of these tests needs to really communicate that to you that there is that risk of a false positive or a false negative. I mean that's something that you have to act upon that you. You have to make that informed choice about whether or not you're willing to accept that.”
Limited understanding of scope of testing	*Rural*
	FG2, female “What are these [sex chromosome conditions]?”
*5. Views of disability*	
Challenges of parenting a child with a disability	*Metropolitan*
	FG1, male “Down syndrome people are amazing and they're beautiful and very lovely. But you know, whether you can have it at that time in your life, maybe there's some people who can't.”
	Rural
	FG 2, female “It would sting for the majority of people, but also the long‐term effects of having child with a disability would also sting and cost a lot more”
	FG2, female “I was like ohh a baby's a baby, I'm happy, I'm good, it doesn't. matter, whereas now I'm like, OK, there's complexities around costs, what I have to do, all the flow on effects like if there are pretty significant disabilities, then, that would be something that I would factor in”
	FG2, female “No, no, I think I would just be really, really shocked. Disappointed. Is that a bad word to use? We do have family members who have quite a significant disability and I can see how much pressure it puts on my fairly old parents at the moment, and we don't want to go through that so, based on your story, I would potentially abort a healthy baby with these results.”
**Values**	
*6. Prioritisation of NIPT*	
Differing values influencing NIPT decision‐making between metropolitan and rural communities	*Metropolitan*
	FG1, female “[In response to another participant stating they would pay for NIPT] Yeah, I think yes, we can cut down on other things, because the well‐being [of the child] is very important.”
	*Rural*
	FG2 female “I was 22 when I had my first one and I didn't do any screening. I was healthy, I was young, I was fairly confident it was gonna be fine” FG2, female “[response] I agree with you, with that like if you've got genetic histories and you have got some sort of disabilities where it could be diabetes, whatever. Like, if you know one that's in your family history, then yeah, I would agree with you. You know to get all the tests. You know, possibly down, no matter how much it's gonna cost me.” FG2, female “[response] Yes, that's the attitude, yeah.”
	FG2, female “Yeah, like, so I'm 12 weeks pregnant. So we're just going through the whole process as well. So my husband and I discussed, and we've decided that we would probably do the old method where we do that first line test. Because the cost of an NIPT at the moment is $420.00. So that's still a lot of money for us and you know we're we're both relatively young.”

Abbreviations: FG; focus groups, NIPT; non‐invasive prenatal testing.

### 3.3 Attitudes

#### 3.3.1 Access to Health Care Providers and Services

##### 3.3.1.1 Variable Impact of Cost as a Barrier

Metropolitan participants engaged in limited discussion around access, and this discussion was focused on cost. Metropolitan participants did not report cost to be a current barrier, but they recognised it as a potential barrier for others and themselves in the past.

Metropolitan male For our first one, we had the cFTS because we were young and didn't have that much money. Not that we have much right now, but we've had the Harmony test [one commercially available NIPT test] for last two.

In the rural groups, cost was a significant barrier for most participants. Similar to the metropolitan group, they discussed the impact of age and personal circumstances in relation to the cost of testing.

Rural female …so I was really sick, and I hadn't been really working, so cost would be huge for us.

##### 3.3.1.2 Wait Time Was a Bigger Concern for Those Likely to Experience Longer Delays

Metropolitan participants did not consider wait times to be an issue affecting them, whereas it was a very important consideration in the rural groups. Several rural participants felt waiting for a result would cause them anxiety and others said wait times would force them to travel to a major city.

Rural female Cause if I had to wait like a week or 2 weeks [for the results], I think I would go mental with anxiety.

Rural female Imagine if that was 6 weeks

Rural female That would be torture

##### 3.3.1.3 Travel to Testing: Rural vs. Metropolitan Challenges

Both metropolitan and rural participants did not want to travel far to access testing, however the definition of ‘far’ varied between the groups. Within rural groups, travel to testing was associated with a number of additional costs.

Metropolitan female If it's 30 minutes. Maybe not.

Rural female It's a huge problem for anyone that's not living in a metropolitan area. It's not just $420, it's a few days off work, accommodation, travel costs.

#### 3.3.2 Delivery of Information and Results

##### 3.3.2.1 Divergent Opinions on How to Receive Information

Across both metropolitan and rural groups, opinions varied on how to receive information, with some favouring in person, telehealth, or email. Some of the rural participants expressed greater concerns about wait times. Most of this discussion centred on the process of receiving results.

Metropolitan male I think good news you take anyway (laughs). But bad news you take in person.

Rural female I'd be calling being like I don't care, give me a telehealth appointment. Like, fit me in 5 seconds. Like they've just gotta, they've just gotta tell you exactly what's on their little piece of paper.

Rural female As quick as the information can come to me, I don't care how blunt it is, or how it's given.

##### 3.3.2.2 Personal Preference Determines Health Professional Type

Participants from both metropolitan and rural groups had differing views on which healthcare professional should deliver the pre‐test information and results. All participants were unaware of genetic counsellors, with some participants making negative assumptions about their role.

Metropolitan female [In response to asking if participants had heard of a genetic counsellor] Before today? No, no I hadn't.

Metropolitan male Genetic specialist. I mean, it sounds like very expensive, but also sounds like they're not you know, trained in other ways

Within the rural groups, the choice of healthcare professional appeared to be driven by cost and wait time.

Rural female It would obviously depend on you know the costs involved with seeing that person, distance to travel, all that sort of thing as well. I highly doubt in a regional area, you're gonna have an enormous supply of those guys [genetic counsellors] hanging around.

#### 3.3.3 Pros and Cons of Expanded NIPT

##### 3.3.3.1 Increasing Information Paradox: Reassurance Versus Uncertainty

Several participants preferred a broader scope of conditions, despite reduced accuracy, as they felt more information provided increased reassurance. Conversely, other participants felt that the decreased accuracy resulted in greater uncertainty. Within the rural group, there was an in‐depth discussion about the anxiety that would be caused by the decreased accuracy.

Metropolitan male I think testing for everything is good because even if it's not 100% accurate all of them, it's worth it right? At least it's better to know a little bit more possibility as opposed to not knowing it.

Rural female So is it like a catch 22. Do you want to know more about your child to get those proper positive results or the chances of the negative sides of the testings.

### 3.4 Beliefs

#### 3.4.1 Misunderstanding and Lack of Awareness

##### 3.4.1.1 Misrepresentation of NIPT as a Diagnostic Tool

Across all focus groups, participants with lived experience (either currently pregnant, or previously pregnant) referred to NIPT as the *‘Harmony test’* (a commercial brand of NIPT) and were under the impression it was diagnostic.

Metropolitan male We've had the harmony test for last two. Like if we did have any positive results, it would not have changed us wanting to have the babies, but with the harmony test we've been told it was conclusive.

Rural female I would potentially abort a healthy baby with these [positive screening] results.

##### 3.4.1.2 Limited Understanding of Scope of Testing

Across all focus groups, most participants were only familiar with Down syndrome and did not appear to have knowledge of the other conditions. One participant believed the prenatal screening they underwent was only for Down syndrome, even though it likely included the other two common trisomies.

Metropolitan male From our experience the test was done just for Down syndrome… but the other ones, I've never heard of them, to be honest, and like short height and the ovaries like that [Turner syndrome], to me is not really that bad.

Rural female I think I asked about it and they said you're getting tested for the genetic stuff and I'm guessing they just gave me the basic one.

#### 3.4.2 Views of Disability

##### 3.4.2.1 Challenges of Parenting a Child With a Disability

Participants in both groups expressed acceptance of children with disabilities, whilst acknowledging the belief that raising them presents potential challenges. Within the metropolitan group, participants raised the issue that some people might not feel able to raise a child with a disability, although none of them identified themselves that way. In the rural focus groups, participants discussed the challenges relevant to them.

Metropolitan male We adopted a child because the family couldn't afford it, because they had like three children and got told it's healthy, but it wasn't.

Rural female It's hardest now just to pay rent and groceries. It wouldn't be fair on the child to, you know, bring that child in and knowing that I won't be able to afford the needs, the travel, the appointments to get that child, what that child needs when it arrives.

### 3.5 Values

#### 3.5.1 Prioritisation of NIPT

##### 3.5.1.1 Differing Values Influencing NIPT Decision‐Making Between Metropolitan and Rural Communities

In the metropolitan group, participants valued choosing NIPT as a way to act in the best interests of their child. On the other hand, rural participants, who faced access barriers, valued NIPT based on their perceived chance of having a child with a chromosome condition. This concept of perceived chance and how it related to the value of NIPT was not raised by metropolitan participants.

Metropolitan female Even I am in favour of testing for as much as possible, because that would be ultimately it's in the interest of the child. So as much as possible, I think testing.

Rural female Now, if I were to have another child. I'm in my late 30s, I'm higher risk, I'll be doing screening and testing.

## 4 Discussion

These focus group results provide evidence regarding Australian public attitudes, beliefs and values around prenatal screening. Both metropolitan and rural groups exhibited similarities, including a desire to undergo prenatal screening and misconceptions around the screening tests available and the conditions assessed. The desire to undertake screening occurred within a social context where participants demonstrated acceptance towards individuals with disabilities whilst acknowledging potential challenges of raising a child with a disability. Our findings suggest there are differences in attitudes between people living in metropolitan versus rural locations, particularly with respect to the different elements of access to screening (cost, wait times and distance).

Metropolitan and rural participants differed in their attitudes towards access. The cost of NIPT was a major barrier for rural participants, whilst metropolitan participants did not perceive this to be an issue given their financial circumstances. Concerns over inequities of access due to the relatively high cost of NIPT between families with high and low SES have been described internationally [8, 19] and in Australia [11]. This is in line with the results of this study, as rural families generally have lower SES [20]. This study adds to the literature by identifying additional inequities related to geographical location. In contrast to metropolitan participants, rural participants expressed concerns about wait times for accessing GP appointments, ultrasounds and follow up testing, as well as the need to travel to access these services. Additional costs associated with travel were raised by rural participants, including the cost of transport, accommodation and loss of income. International qualitative studies have found wait times to be a common concern but differences by geographic location have not been explored [8, 21, 22]. Discussions are limited within the literature regarding the distance travelled to access prenatal screening. This study highlights the multifaceted inequities faced by rural communities in accessing prenatal screening services.

The current study finds divided views on expanding the scope of conditions included in prenatal screening, with no clear differences between metropolitan and rural groups. Two models of thought became apparent, one in which additional information offered reassurance, and another where it heightened uncertainty and stress, consistent with focus groups conducted in Canada [8]. In Australia, clinicians are worried about the psychosocial harms and ethical concerns resulting from expanded NIPT, especially when screening for sex chromosome conditions and microdeletion and microduplication syndromes [11, 12]. These screenings are linked to high rates of false positives and variable phenotypes, which can further complicate the interpretation of the results. Our findings indicate that whilst several participants supported broadening the scope of testing, many lacked understanding regarding the conditions that can be screened. This contrasts to previous Australian studies, where people who have undergone NIPT have generally reported being satisfied with pre‐test information [9, 23]. This raises questions about whether pregnant couples are truly providing informed consent and making informed decisions. Traditionally, consent discussions have focused on procedural risks, rather than the potential outcomes of the test, as observed with invasive prenatal diagnostic procedures. However, with NIPT, the minimal procedure‐related risk removes this traditional barrier and contributes to its routinisation. It has been suggested that the routinisation of testing shortens the decision‐making process, creates an expectation for pregnant people to undergo testing and minimises the perceived importance of these decisions [24]. Similar concerns have been raised in health systems that publicly fund NIPT, where it is perceived as a routine test with no cost, meaning there is no friction causing people to think about the gravity of their decision and possible implications [8].

The current study indicates the Australian public may not prioritise the method by which they receive information, even in the face of existing challenges related to information access. This observation was seen across the type of healthcare professional and mode of communication, during the delivery of pre‐test information and test results. Contrary to expectations, there were mixed attitudes towards genetic counsellors, with a lack of awareness and some negative assumptions about the profession. This is consistent with an international quantitative study on preferences for prenatal diagnosis, which found women were indifferent between receiving results from a genetics specialist or non‐specialist [25]. It is important to note that clinicians and policymakers view genetic counsellors as capable of providing higher quality support, and in Australia, they are recommended for individuals receiving high‐chance results [26]. Our study reveals varied opinions on the preferred mode of receiving information. These findings align with a recent international quantitative online survey, which found women preferred information to either be text‐based, in person, or through an app, but not in a group discussion [27]. Interestingly, recent Australian evidence within a socioeconomically disadvantaged population found different forms of counselling for prenatal screening did not have significant differences in effectiveness [28]. An additional finding from our study was participants with lived experience of NIPT believed it to be a diagnostic test, and those who were pregnant found it challenging to obtain information.

The introduction of NIPT may have implications for people living in rural locations in accessing prenatal screening. Whilst the cost of NIPT may be a challenge for these communities, NIPT could actually mitigate the need for extensive travel as only a blood test is required (whilst cFTS requires an ultrasound), and there are fewer unnecessary diagnostic tests [3]. This supports the argument for public funding for NIPT based on geographic location. To improve understanding of the test and test results, there should be a focus on (1) the information provided by the test and (2) information dissemination. First, ensuring the test information itself is improved, for example, by excluding conditions with very low positive predictive values. Second, ensuring clear information is widely available. Since the type of healthcare provider does not appear to be important, alternative providers such as practice nurses could be trained to provide this information. Although this would require significant investment, it would act as a protective buffer against misconceptions around prenatal screening. By employing these strategies, genetic counsellors could then be prioritised for those who receive high‐chance results, whilst addressing potential negative attitudes towards the profession. The use of digital technologies could also be explored to improve access in rural areas.

### 4.1 Limitations

This research explores patterns and overall views of members of the general population from NSW but does not represent every individual in metropolitan and rural/regional areas across Australia. For instance, the cost of NIPT is likely to be a barrier for some pregnant people and partners in metropolitan locations, and people living in remote or very remote areas in Australia may have different views. Although we aimed to explore the views of females and males, most participants were female. The sample size was deemed sufficient to identify key themes and provide illustrative examples. However, a larger sample size could have facilitated a deeper understanding of these themes and ensured data saturation. As part of the information collection prior to the focus groups, we included questions on lived experience. However, this question was not completed by many participants; instead, it emerged organically during the discussions. We excluded people who could not speak English, who are likely to experience additional barriers in accessing prenatal screening and who are also likely to be members of culturally and linguistically diverse communities who in turn are likely to have different perspectives to the study participants.

## 5 Conclusion

In the absence of public funding and a nationally coordinated approach to the implementation of NIPT in Australia, we have identified concerns regarding inequities in access to NIPT, the extent to which pregnant people and their partners understand the purpose of NIPT (i.e. screening, not diagnosis), and the implications of these for parental decision‐making in the antenatal period arising especially in the context of tests with different scope (i.e. three common trisomies only versus expanded testing for other conditions). Our findings highlight that different policy approaches are needed to improve access to NIPT for people living in rural areas, to ensure informed consent for NIPT, and that the pre‐test information provided to pregnant people and their partners clearly accounts for the range of public perspectives observed across different geographic locations.

## Conflicts of Interest

The authors declare no conflicts of interest.

## Supporting information


Data S1.

Data S2.

## Data Availability

The data used in this study is accessible upon reasonable request from the corresponding author, in accordance with the confidentiality agreements and ethical considerations governing the research. Requests for access to the data should be directed to Amber Salisbury at amber.salisbury@sydney.edu.au.
