# Supplementary material for: Public Perspectives Around Prenatal Screening of Chromosomal Abnormalities: A Focus Group Study Comparing Metropolitan and Rural/Regional Areas in Australia
Source: Aust N Z J Obstet Gynaecol. 2025 Feb 7;65(4):506–17. doi: 10.1111/ajo.13935 (PMC12668888; doi:10.1111/ajo.13935)
Supplement: Supplementary file 1 — Data S1. [file AJO-65-506-s002.docx]

# Supplementary Material

# Rapid literature review

A rapid literature review was performed in the University of Sydney library to understand the broad scope of factors which may impact on preferences during prenatal screening. The search was limited to systematic reviews and used search terms similar to the following: “prenatal testing”, “non-invasive prenatal testing”, and “preferences”. A recent systematic review (2021) was identified which explored quantitative or mixed-methods studies examining factors that affect decision making processes during prenatal testing.^1^

The systematic review included 46 studies in their qualitative synthesis. A member of the research team collated and compared the factors within the included studies. A total of 54 factors were identified and have been classified into the following groups: (1) outcomes; (2) personal; (3) process; (4) psychosocial; and (5) test (Table 1).

*Table 1 Factors from the literature which may impact on parental decision making in prenatal testing*

| **Factor type** | **Factor** |
| --- | --- |
| 1. Outcomes | False negative cases |
|  | False positive cases |
|  | Fertility treatment |
|  | Quality of life |
|  | Social impact |
|  | Uncertain results |
| 1. Personal | Acceptibility of having a baby with DS |
|  | Accurate recall of risk estimates |
|  | Clinical characteristics |
|  | Family history |
|  | History of spontaneous miscarriage |
|  | Intention to participate in screening |
|  | Knowledge of Down syndrome |
|  | Knowledge of screening tests |
|  | Knowledge of invasive testing |
|  | Knowledge of intentions of outcome of pregnancy based on test results |
|  | Maternal age |
|  | Perceived risk of having a baby with DS |
|  | Perceived risk of PRL |
|  | Personality |
|  | Previous child with illness |
|  | Relational factors |
|  | Risk taking |
|  | Sociodemographic characteristics |
|  | Understanding of screening results |
|  | Values, beliefs and attitudes |
| 1. Process | Available public funding |
|  | Convinience of procedure |
|  | Cost |
|  | Decision making process |
|  | Impression of clinician's preference |
|  | Information provision |
|  | Method or receving results |
|  | Method of risk reporting |
|  | Satisfaction with decision making process |
|  | Test location |
|  | Time to make a decision |
|  | Timing of genetic counselling |
|  | Type of healthcare provider delivering results |
|  | Wait time for results |
| 1. Psychosocial | Anxiety |
|  | Decisional conflict |
|  | Decisional regret |
|  | Depression |
|  | Percevied choice control |
|  | Personal wellbeing |
|  | Pregnancy stress |
|  | Psychosocial factors |
|  | Regret |
|  | Worries in pregnancy |
| 1. Test | Miscariage risk |
|  | Sensitivity |
|  | Specificiy |

# Discussion guide

*Table 2 Discussion guide*

| **Section** | **Description** | **Key questions** | **Prompts** |
| --- | --- | --- | --- |
| Warm up1 – ice breaker | Warming up the participants by getting them to introduce themselves and establishing a relaxed environment | Can everyone please introduce themselves | What is your name and what do you spend most your time doing? |
| Section 1 | Discuss each feature on the list, with reference to the levels, and to the wording of features and levels | What does [feature/level] mean to you?  What wording would make the features or levels easier to understand?  Which of these are important to you and why?  Which features do not seem important to you and why? | How would feature X influence your decision to take part in the screening program? |
| Section 2 | Groups will be asked to discuss the ranking of features and then to individually rank the features after the discussion using an online form. | How would you order the features from most to least important? | Which feature ranking do you not agree with?  Which feature do you think is most important?  Which feature do you think is least important? |
| Final question | Last question to gather any extra information | What are your final thoughts on the on the different ways of ranking the features?  How were people’s opinions changed through the discussion?  Is there anything else you would like to add to this discussion? | What changed your mind? |

1. Di Mattei V, Ferrari F, Perego G, et al. Decision-making factors in prenatal testing: A systematic review. *Health Psychology Open* 2021; 8: 2055102920987455-2055102920987455. DOI: 10.1177/2055102920987455.
