# Supplementary material for: Public Perspectives Around Prenatal Screening of Chromosomal Abnormalities: A Focus Group Study Comparing Metropolitan and Rural/Regional Areas in Australia
Source: Aust N Z J Obstet Gynaecol. 2025 Feb 7;65(4):506–17. doi: 10.1111/ajo.13935 (PMC12668888; doi:10.1111/ajo.13935)
Supplement: Supplementary file 2 — Data S2. [file AJO-65-506-s001.pdf]

Prenatal screening

Amber Salisbury

THE UNIVERSITY OF SYDNEY

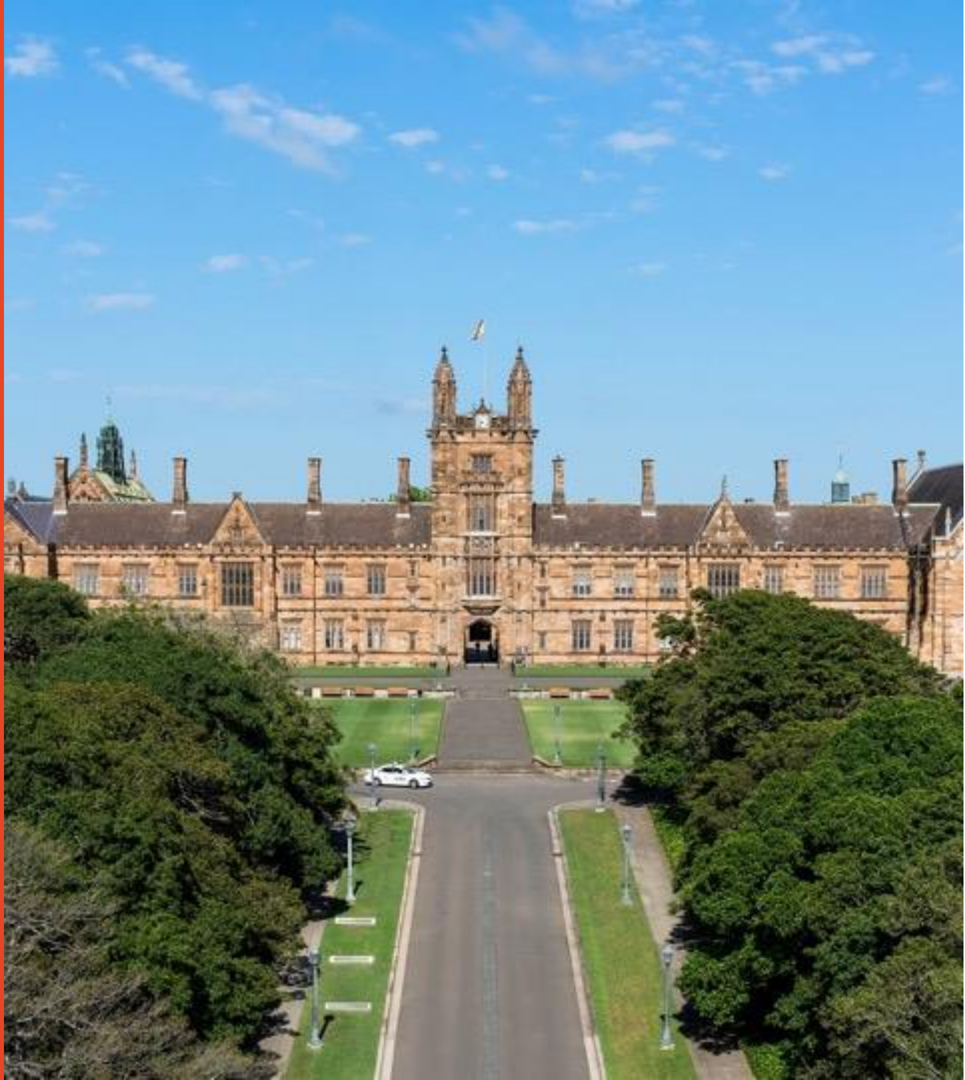

Acknowledgment of country

We would like to acknowledge and pay respect to the traditional owners of the land on which we meet; the **Gadigal people of the Eora Nation**. It is upon their ancestral lands that the University of Sydney (Camperdown Campus) is built.

As we share our own knowledge, teaching, learning and research practices within this University may we also pay respect to the knowledge embedded forever within the Aboriginal Custodianship of Country

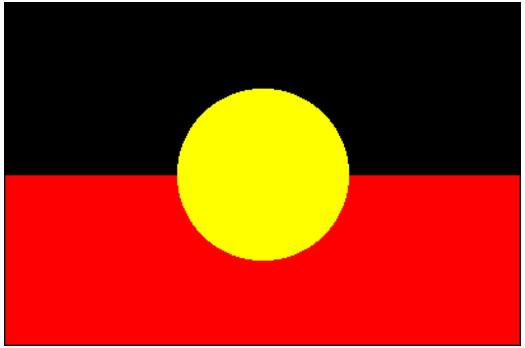

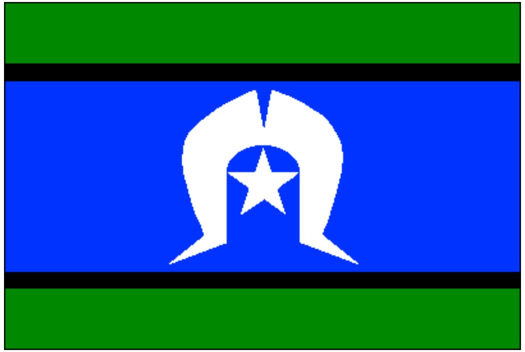

Prenatal screening

- Combination of tests to see if your unborn baby is more or less likely to have one of these conditions *(reported as a low- or high-risk result)*
  - **Down syndrome** (Trisomy 21)
  - **Edwards syndrome** (Trisomy 18)
  - **Patau syndrome** (Trisomy13)
  - Sex chromosome conditions (e.g. Turner syndrome)
  - Other rare conditions
- If you get a high-risk screening result, you will be offered a diagnostic test to confirm the screening results
- Diagnostic tests are invasive and have a small risk of miscarriage (0.2-0.3%)
- *The screening test can also determine the sex of the unborn baby*

https://www.youtube.com/watch?v=PfRJe8Wxkew

Types of screening tests

Combined first trimester screening (cFTS - conventional screening)

- Blood test and ultrasound
- Screens for common trisomies and congenital conditions

Neither test is diagnostic

Non-invasive prenatal testing (NIPT - recently introduced)

- Blood test (of DNA from the placenta, that is in the mother's blood)
- More accurate than cFTS in detecting common trisomies
- Can also screen for sex chromosome conditions and other rare conditions, but with lower accuracy
- Can be offered as first-line or second-line testing
  - Ultrasound also required
- *Can also determine the sex of the unborn baby*

Scenario 1 – low risk screening results

Pathway	Value
cFTS timing	12 weeks
cFTS results	Low risk for common trisomies
NIPT timing	NA
NIPT result	NA
Invasive test timing	NA
Invasive test result	NA

0.3% chance this is a false negative

This means it looks as if the baby does not have one of the conditions, when actually it does.

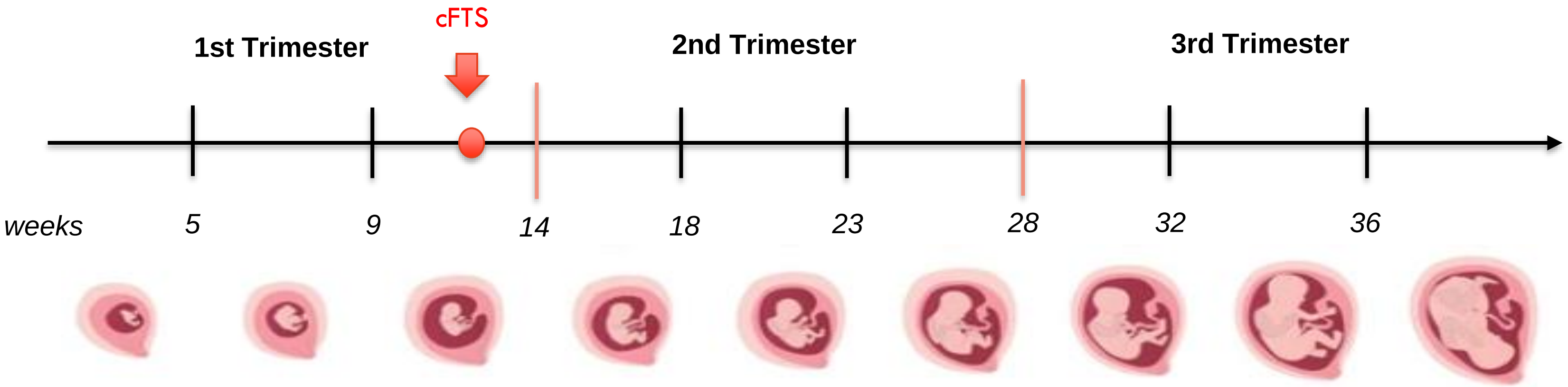

Scenario 2 – high risk screening results (T21)

Pathway	Value
cFTS timing	12 weeks
cFTS results	High risk for Down syndrome
NIPT timing	14 weeks
NIPT result	High risk for Down syndrome
Invasive test timing	15 weeks (CVS or Amnio)
Invasive test result	Normal result

This means it looks as if the unborn baby has one of the conditions, but actually it doesn't

False positive

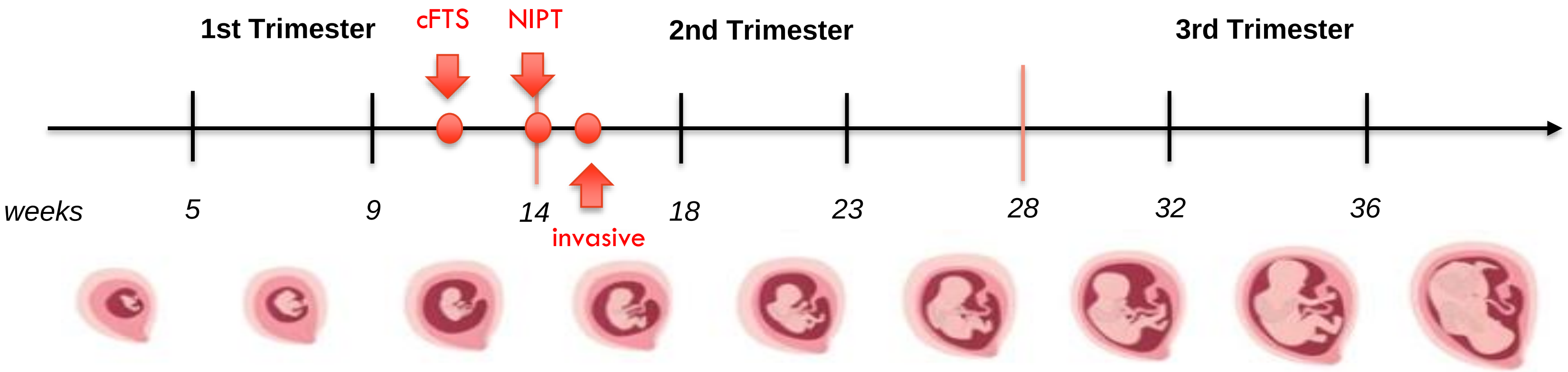

Scenario 3 – high risk screening results (sex chromosome conditions)

Pathway	Value
cFTS timing	NA
cFTS results	NA
NIPT timing	11 weeks
NIPT result	High risk for Turner syndrome
Ultrasound timing and result	13 weeks, normal
Invasive test timing	16 weeks (Amnio)
Invasive test result	Turner syndrome diagnosis (45, X)

Added for first-line NIPT

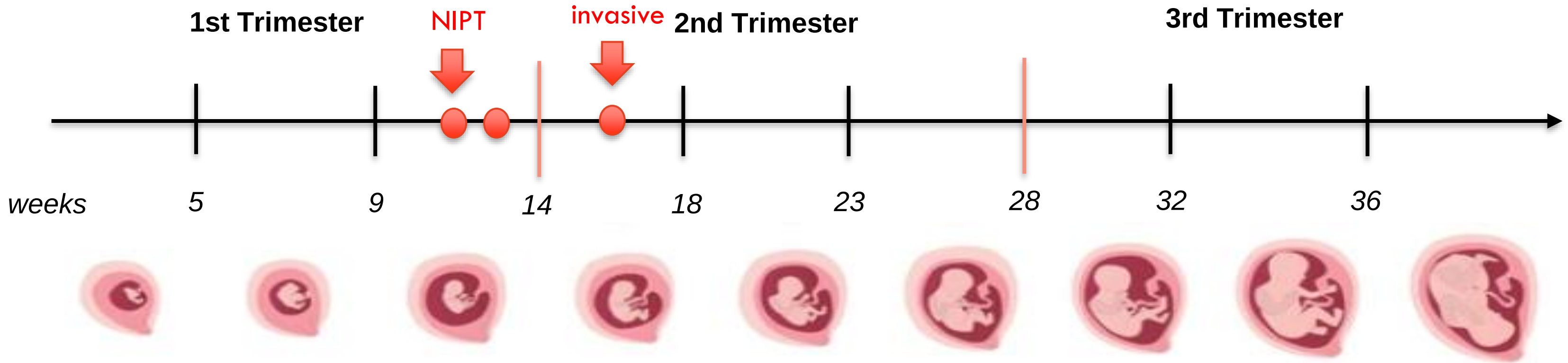

Scenario 4 – high risk screening results (T18)

Pathway	Value
cFTS timing	12 weeks
cFTS results	Low risk for common trisomies, abnormal ultrasound
NIPT timing	12 weeks
NIPT result	High risk for Edwards syndrome (T18)
Invasive test timing	13 weeks (CVS)
Invasive test result	Edwards syndrome diagnosis (T18)

False negative

This means it looks as if the baby does not have one of the conditions, when actually it does.

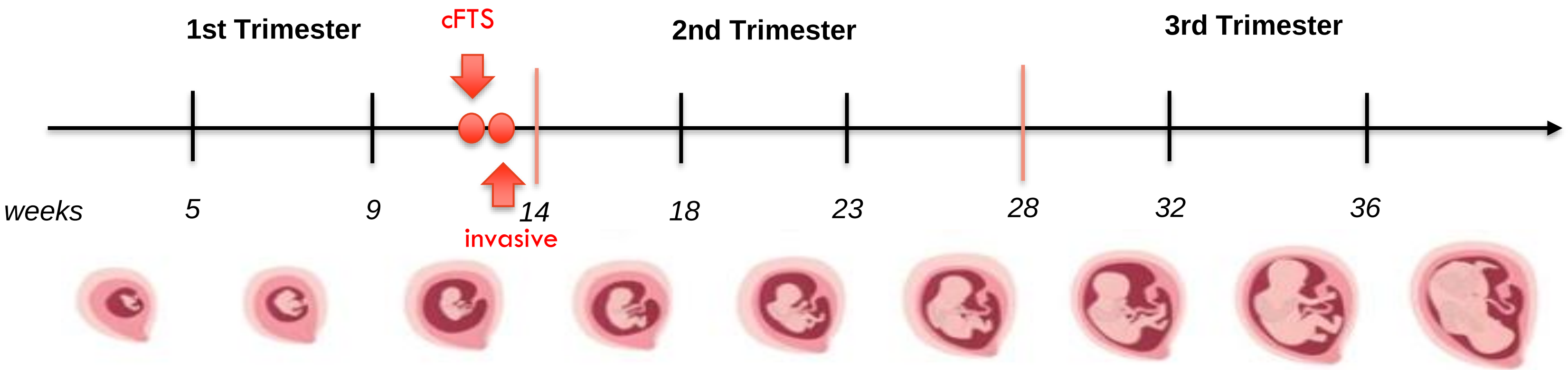

What it's like to receive an unexpected result

https://www.youtube.com/watch?v=Diz2x3iM5ME

Features to discuss

Features of the test and the test results

- What the test covers (common trisomies, sex chromosome conditions, other rare conditions, sex)
- Number of individuals correctly diagnosed
- False positive result
- False negative result
- Inconclusive result
- Maternal incidental findings

Features of the process of being tested

- Cost
- Wait time
- Travel distance to appointments
- Who provides information before the test is done
- How the test results are delivered
- Who delivers the test results

Extra info

- Edwards syndrome is a genetic condition in babies that causes severe disability. It is caused by an extra copy of chromosome 18 and babies born with the condition usually do not survive for much longer than a week.
- Patau syndrome causes severe intellectual disability and physical defects. Most infants with this condition don't live past their first week of life. Treatment varies from child to child and focuses on relieving symptoms and managing complications.

Extra info

- Turner syndrome, a condition that affects only females, results when one of the X chromosomes (sex chromosomes) is missing or partially missing. Turner syndrome can cause a variety of medical and developmental problems, including short height, failure of the ovaries to develop and heart defects.

Extra info (T21 only)

cFTS

- Detection rate: 79%
- False negative rate: 21%
- False positive rate: 5.4%

NIPT

- Detection rate: 100%
- False negative rate: 0%
- False positive rate: 0.1%
- Inconclusive rate: 3%

Not as accurate for other conditions!!
